# Supplementary material for: Empirical methods for controlling false positives and estimating confidence in ChIP-Seq peaks
Source: BMC Bioinformatics. 2008 Dec 5;9:523. doi: 10.1186/1471-2105-9-523 (PMC2628906; doi:10.1186/1471-2105-9-523)
Supplement: Additional File 1 — A variety of html documents from the USeq web site detailing the available applications, their best usage, output file type descriptions, command line menus, etc. [file 1471-2105-9-523-S1.zip › USeqUserGuides/JavaDoc.zip!/JavaDoc/util/apps/Flash.html]

Flash


|  |  |  |
| --- | --- | --- |
| **Overview** \| **Package** \| **Class** \| **Use** \| **Tree** \| **Deprecated** \| **Index** \| **Help** | |  |
| **PREV CLASS**   **NEXT CLASS** | **FRAMES**    **NO FRAMES**     **All Classes** |  |
| SUMMARY: NESTED \| FIELD \| CONSTR \| METHOD | DETAIL: FIELD \| CONSTR \| METHOD |  |


---


## util.apps Class Flash

```
java.lang.Object
  util.apps.Flash
```

---

``` public class Flash extends java.lang.Object ```

---

**Constructor Summary**

`Flash(java.lang.String[] args)`


| **Method Summary** | |
| --- | --- |
| `void` | `addFlashCard(util.apps.Flash.FlashCard card)`             Adds flash card to the end of the array. |
| `void` | `interrogate()` |
| `static void` | `main(java.lang.String[] args)` |
| `util.apps.Flash.FlashCard[]` | `parseFile(java.io.File file)`             Strips out " that stupid excel adds in. |
| `void` | `queryOrder()` |
| `void` | `queryShuffle()` |
| `void` | `shuffleCards()`             Randomly permutates the FlashCard[]. |

**Methods inherited from class java.lang.Object**

`equals, getClass, hashCode, notify, notifyAll, toString, wait, wait, wait`

**Constructor Detail**

### Flash

```
public Flash(java.lang.String[] args)
```


**Method Detail**

### interrogate

```
public void interrogate()
```

---


### queryOrder

```
public void queryOrder()
```

---


### queryShuffle

```
public void queryShuffle()
```

---


### addFlashCard

```
public void addFlashCard(util.apps.Flash.FlashCard card)
```

:   Adds flash card to the end of the array.

---


### shuffleCards

```
public void shuffleCards()
```

:   Randomly permutates the FlashCard[].

---


### main

```
public static void main(java.lang.String[] args)
```

---


### parseFile

```
public util.apps.Flash.FlashCard[] parseFile(java.io.File file)
```

:   Strips out " that stupid excel adds in. Skips lines beginning with #


---


|  |  |  |
| --- | --- | --- |
| **Overview** \| **Package** \| **Class** \| **Use** \| **Tree** \| **Deprecated** \| **Index** \| **Help** | |  |
| **PREV CLASS**   **NEXT CLASS** | **FRAMES**    **NO FRAMES**     **All Classes** |  |
| SUMMARY: NESTED \| FIELD \| CONSTR \| METHOD | DETAIL: FIELD \| CONSTR \| METHOD |  |


---
